# Nonlinear wave interactions between short pulses of different spatio-temporal extents

**DOI:** 10.1038/srep29010

**Published:** 2016-07-06

**Authors:** Y. Sivan, S. Rozenberg, A. Halstuch, A. A. Ishaaya

**Affiliations:** 1 Unit of electro-optics Engineering, Faculty of Engineering Sciences, Ben-Gurion University of the Negev, Be’er Sheva, 8410501, Israel; 2 Department of Electrical Engineering, Faculty of Engineering Sciences, Ben-Gurion University of the Negev, Be’er Sheva, 8410501, Israel

## Abstract

We study the nonlinear wave interactions between short pulses of different spatio-temporal extents. Unlike the well-understood mixing of *quasi*-monochromatic waves, this configuration is highly non-intuitive due to the complex coupling between the spatial and temporal degrees of freedom of the interacting pulses. We illustrate the process intuitively with transitions between different branches of the dispersion curves and interpret it in terms of spectral exchange between the interacting pulses. We verify our interpretation with an example whereby a spectrally-narrow pulse “inherits” the wide spectrum of a pump pulse centered at a different wavelength, using exact numerical simulations, as well as a simplified coupled mode analysis and an asymptotic analytical solution. The latter also provides a simple and intuitive quantitative interpretation. The complex wave mixing process studied here may enable flexible spatio-temporal shaping of short pulses and is the starting point of the study of more complicated systems.

Wave mixing is one of the most basic nonlinear optical processes. In the vast majority of cases, it is studied for one or more interacting *quasi*-monochromatic waves, for which all temporal and spatial scales are similar and much larger than the period(s) and wavelength(s), respectively^1^. In contrast, there are very few studies of the mixing of wave packets having different spectral widths, and temporal and spatial profiles^1^,^2^. Indeed, these configurations involve a rather complicated and non-intuitive wave mixing process such that it is difficult to assess, a priory, what would be the final spatial length, temporal duration and spectral width of the pulses generated by the interaction.

In this Article, we present a systematic way to predict and interpret the complex wave interactions in such scenarios in terms of exchange of spectral and spatial Fourier components between the interacting pulses. Specifically, we study the case where a short pulse (e.g., femtosecond or picosecond long) is extracted from a much longer pulse. Such a process is enabled by a wave-mixing interaction we denote as *spectral inheritance* - a temporally long (and spectrally-narrow) pulse propagating in a material with an intensity-dependent refractive index “inherits” the wide spectrum of an intense, temporally short (spectrally-broad) pump pulse centered at a different wavelength.

We focus on a case where the (short) pump pulse is periodically-patterned such that it induces a transient Bragg grating (TBG) whose stop gap matches the frequency of an incoming (long) signal pulse. TBGs have been studied extensively^3^,^4^ mostly in the context of measuring fundamental material properties such as diffusion coefficients and free-carrier recombination time. Almost no attention was given, however, to the possibility of using TBGs for spectral and spatio-temporal shaping of pulses, as well as for using them for telecom applications such as all-optical switching. Indeed, this configuration offers great flexibility in determining the duration and intensity of the reflected pulse and can be adapted to any incoming wavelength by a simple adjustment of the spatial period of the TBG.

Intuitively, the TBG will cause signal photons captured within it to be reflected during its “lifetime”. It is insightful to interpret the wave mixing process induced by the TBG using dispersion curves and transitions between them, see Fig. 1. In general, the TBG causes a (forward propagating) signal photon, characterized by a longitudinal momentum *k*_*f*_ > 0 and *ω*_*f*_ to be converted into a new (backward propagating) photon, characterized by *k*_*b*_ < 0 and *ω*_*b*_. The relation between these quantities is determined by the spatio-temporal spectral content introduced by the perturbation of the optical properties of the waveguide material, 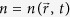. Indeed, a perturbation of the refractive index would give rise to a change of the canonically-conjugate properties of the waves in the system, namely, a change of the refractive index *in space* would cause a change of their *momentum* whereas a change of the refractive index *in time* would lead to a change of their *spectral* content. Specifically, the spatial period of the TBG provides the momentum necessary to couple the forward wave component to the backward wave component, namely, to enable the horizontal transition *k*_*f*_ → *k*_*b*_ = *k*_*f*_ − 2*k*_*f*_ = −*k*_*f*_, *ω*_*b*_ = *ω*_*f*_ (Fig. 1; black arrow). However, the spatial and temporal finiteness of the induced TBG introduces additional momentum and frequency components which enable a *continuum of oblique transitions* in the dispersion plot, so that in general, *k*_*b*_ ≠ −*k*_*f*_ and *ω*_*b*_ ≠ *ω*_*f*_ (Fig. 1; red and blue arrows represent the generation of photons which are red and blue shifted with respect to the signal photons). Thus, the backward pulse can have a substantially wider bandwidth compared with the incoming forward propagating signal pulse.

The necessary index change, 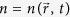, can be imposed in various ways, depending on the constituent materials. For example, in a dielectric material, such as silica, this can be achieved via cross-phase modulation (XPM) between (intense) pump(s) and a signal pulse via the Kerr effect^5^. In this case, one can interpret the spectral inheritance as a 4-photon process whereby an incoming signal photon *ω*_*f*_ interacts with two pump photons with frequencies *ω*_*p*_ ± Δ*ω*_*j*_ such that the new (backward) frequency component is


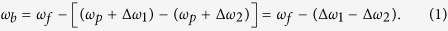


Here, we assumed that the frequency of one pump photon is added to the frequency of the incoming (forward propagating) signal photon whereas the frequency of the second pump photon is subtracted from it. This is in accord with standard XPM processes in Kerr media where the third-order polarization is proportional to


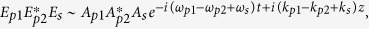


where *A*_*pj*_ and *A*_*s*_ are the amplitudes of the pump and signal waves, respectively^1^. Momentum conservation follows the same relations, however, the momentum components of the pump photons along the waveguide have to be opposite in order to ensure that the total momentum gained by the signal photon is ≈−2*k*_*f*_ such that the idler photon has *k*_*b*_ ≈ −*k*_*f*_. Indeed, this is the case inducing a BG through a phase mask in which case the pump photons are counter-propagating in the direction parallel to the waveguide.

The wave-mixing process (1) describes a single 4-photon interaction in which a part of the pump photon energy (or similarly, momentum) is transferred to or received from the (forward propagating) signal. However, the net frequency change is random, so that on average (i.e., over many such processes), the pump photon spectrum is unaffected. On the other hand, Eq. (1) also shows that the spectrum of the backward pulse is wider than that of the incoming signal - this is the signature of *spectral inheritance*. In fact, the spectrum of the backward pulse can be wider than that of the pump, e.g., when the signs of Δ*ω*_*j*_ are different. Indeed, the cubic interaction corresponds to index modification which is temporally shorter than the duration of each of the pump pulses. This effect is well-known also in second-order nonlinear media as it limits the performance of Frequency-Resolved Optical Gating^6^. Interestingly, since the coupling does not involve an accumulation of phase, unlike Self-Phase modulation interactions^1^, here, the spectral broadening is concomitant with temporal compression.

The wave interactions described so far are in fact general - any multiple wave mixing process can be interpreted in terms of reflection from a grating created by interference of 2 or more waves; at the same time, any such interaction can be interpreted in terms of the frequency and momentum content imposed on the system via 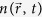. Indeed, spectral inheritance can be implemented in other nonlinear media. Indeed, since the required wave interaction can be *incoherent*, i.e., it does not have to depend on the phase of the pump pulses but rather, only on their intensity, one can exploit slower but stronger nonlinear processes such as free-carrier (FC) generation or thermal nonlinearities, and achieve high efficiencies. In these realizations, energy is absorbed in the material, i.e., the pump photon number is not conserved and the 4-photon interaction picture associated with XPM is not valid. Instead, but still in accord with the interpretation provided in Fig. 1, the dynamic modification of the refractive index caused by the generation of FCs manifests the imprinting of the wide spectral content of the pump pulse on the material constituents (e.g., electron dynamics). This spectral content can then be ‘released’ again in the form of a spectrally-wide backward pulse. In this context, two merits of our approach have to be emphasized. First, although the slow response of FCs and thermal nonlinearities (several 10s of picoseconds or much slower) is usually regarded as a limitation, in the current context, such nonlinearities can be exploited to generate backward pulses of several tens or even hundreds of picoseconds, as shown below; these pulse durations are otherwise difficult to access. Second, it was recently shown by Sivan *et al.* that when a *short* pump pulse is used to generate a TBG of FCs, this grating can (self-)erase on deep sub-picosecond timescales due to FC diffusion, enabling high switching efficiencies and speeds^7^,^8^.

The rest of this Article is dedicated to a comprehensive quantitative study of the phenomenon of spectral inheritance and its limitations. We begin with a Kerr-based example that validates the *qualitative* graphical interpretation of spectral inheritance presented above. We then move on to a *quantitative* study of the phenomenon, and to additional examples based on slower nonlinearities. Throughout the Article, we use terminology appropriate for optical switching of electromagnetic waves at optical frequencies, however, the same concepts apply also for other parts of the spectrum, for electronic switching as well as for waves in many other systems, including e.g., spin waves^9^, acoustic waves^10^ and water waves^11^.

In order to demonstrate spectral inheritance in a realistic and simple system, we initially consider the case in which a signal wave is propagating in a single-mode slab (invariance to one transverse coordinate) waveguide with a Kerr response which is pumped transversely by a spatially-patterned short pump pulse (implemented by two obliquely interfering pulsed beams), see Fig. 2. This configuration enables good uniformity of the refractive index change, both in Kerr media as well as for FC generation^12^/thermal gratings^4^ and is technologically compatible with existing commercial systems. The index change pattern induced by the pumps creates a TBG that spatially overlaps the signal and which is tuned to half the effective wavelength of the signal wave. The pump wavelength should be shorter than that of the signal such that the necessary periodic pattern can be created by the pumps with no violation of the diffraction limit. This is, in fact, the standard case for optical switching semiconductor systems. This also facilitates the spectral distinction between the backward pulse and remnants of the pump light which are partially trapped in the waveguide. We emphasize that this pumped waveguide configuration does not limit the generality of the discussion - it is merely an example of a general wave mixing occurring in a confined region of space.

Figure 3 shows the results of an *exact FDTD* numerical solution of Maxwell equations for a silica waveguide configuration using the commercial software package Lumerical Inc. Indeed, it is seen that the backward pulse can be substantially shorter than the incoming signal, as well as shorter than the pump duration, thus, unambiguously demonstrating *spectral inheritance*. Importantly, our simulations show the same behaviour even for very intense signals whose power *exceeds* that of the pump pulse, i.e., the signal need not be weaker than the pump.

Having established the validity of the concept and the graphical interpretation based on the dispersion relations, we now would like to improve our quantitative understanding of the phenomenon. Figure 3 also shows a complex dependence of the backward pulse efficiency and duration on the various parameters which is difficult to predict a-priory. Thus, in order to explain these observations and in order to simplify the numerical calculations, we derived a simpler theoretical model and solved it analytically. As mentioned, our model relies on an extension of standard (i.e., purely spatial) coupled-mode theory (CMT)^13^ to pulse propagation and time-dependent perturbations^8^. The derivation of this model is motivated by the interpretation of spectral inheritance in terms of a spatio-temporal perturbation of the refractive index of the waveguide, such that the perturbation is given by 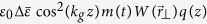, where 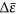 is the maximal index change, *m* is the temporal profile of the perturbation, *W* is its transverse spatial profile and *q* is its longitudinal spatial profile of characteristic length L^*^. In the absence of coupling to any other mode (e.g., for single mode waveguides) and assuming dispersion effects are manifested only on length-scales longer than all other relevant ones (assumptions which are validated by the exact FDTD simulations, Fig. 3), the evolution of the forward (*A*_*f*_) and backward (*A*_*b*_) pulse amplitudes is given by


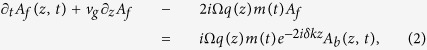



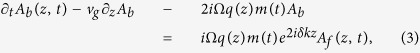


where Ω ≡ *v*_*g*_*κ*, *v*_*g*_ is the group velocity, *κ* is the coupling coefficient, given by the product of the magnitude of the perturbation, 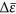, and the spatial overlap of the transverse profiles of the modes and the exact transverse spatial profile of the perturbation *W*)^7^,^8^. In addition, *δk* ≡ *δω*/*v*_*g*_ where *δω* is the detuning of the signal frequency from the center of the induced stop-gap.

An exact solution of Eqs (2 and 3) is possible only numerically. Figure 3 shows that the solutions of the CMT Eqs (2 and 3) are in excellent agreement with the exact solutions of the Maxwell equations. The small discrepancies originate from e.g., the gradual turning-on of the TBG in the transverse direction and the phase distortion (see the third terms on the LHS of Eqs (2 and 3)). We would now want to improve our understanding of the underlying wave physics by obtaining an approximate analytical solution for Eqs (2 and 3). One way to do that is to ignore the spatial derivatives (justified for short perturbations during which the pulse is nearly stationary) - this yields the well-known Rabi solution, see e.g., [13, p. 568], which was shown to give a reasonable accuracy in measurements with spin waves^15^. An alternative analytical solution of Eqs (2 and 3) can be obtained in the low conversion efficiency limit, without neglecting the spatial derivatives. In this case, following^7^,^14^,^16^,^17^, we get 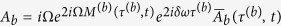, where


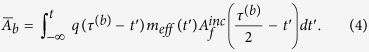


Here, *τ*^(*b*)^ ≡ *z*^(*b*)^/*v*_*g*_, 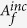 is the incident signal profile, 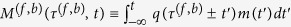 represents the spatio-temporal phase imprinted on the signal due to its interaction with the pump and where the effective perturbation 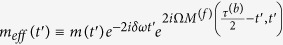 accounts for XPM, absorption and possible detuning. Note that for long switched spots (*q* ≈ 1 or *L* → ∞) and a short (non-adiabatic) perturbation, the backward wave is a time-reversed replica of the forward wave, as demonstrated theoretically^14^,^16^,^17^ and experimentally^18^.

Here, however, we focus on another limit, in which the forward wave is (nearly) monochromatic, i.e., 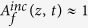, in which case,


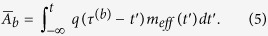


The explicit time-dependence disappears in the limit *t* → ∞ where the perturbation is essentially complete and the backward pulse is given by a convolution of the “temporal” shape (via *m*(*t*)) and spatial shape (via *q*(*z*)) of the induced grating. The expression (5) represents in a transparent manner the unusual coupling of the (transverse) spatial degrees of freedom and temporal degrees of freedom of the pump pulse occurring in our configuration; the latter are related to the longitudinal spatial degrees of freedom of the pump pulse. This simple form can be exploited as a novel approach for pulse shaping manipulations.

We now denote the time it takes a photon to cross the TGB by *T*_*pass*_ ≡ *L*/*v*_*g*_ and the effective switching time by *T*_*sw*_ (As mentioned above, the switching time can differ from the pump duration due to the cubic nature of an instantaneous Kerr nonlinearity, due to the delayed nature of a FC or thermal nonlinearity etc.). Thus, Eq. (4) shows that once the grating is turned *on*, photons within the grating get reflected. If then the TBG is turned *off* immediately after being turned on, i.e., if 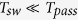, then, the spatial length, hence, the duration of the backward pulse is given by *T*_*pass*_ - the perturbation generates a snapshot of the switched signal segment. Indeed, in this case, *A*_*b*_ ~ *q*(*z* − *v*_*g*_*t*). In this sense, spectral inheritance is minimal - not all the spectral content of the pump is transferred to the backward wave. The reflection efficiency, however, will scale with *T*_*sw*_ (i.e., the shorter time-scale). Somewhat counter-intuitively, in this case the efficiency does not depend on the number of periods in the grating (i.e., on *L*)! *L*, on the other hand, determines the *duration* of the reflected pulse. Heuristically, this occurs because the photons captured within the TBG do not have time to reach its edges, so that in a sense, they “feel” an infinitely-long grating and the dependence of the reflection efficiency on the grating length is “saturated”. This case can be referred to as the (spatially) long grating limit or (temporally) short pump limit. As the pump duration is increased, backward photons are continuously getting generated within the grating, giving rise to a longer backward pulse described by the convolution-type result (5).

In the opposite limit, the spatially short grating will reflect photons during its (long) lifetime so that *A*_*b*_ ~ *m*(*z* − *v*_*g*_*t*). As the spatial extent of the TBG is increased, photons generated at different spatial locations within the grating build up spatially and temporally longer pulses. Here, spectral inheritance is optimal (*T*_*sw*_ is the duration of the backward pulse) and the efficiency is determined by *T*_*pass*_, as expected in the permanent grating limit. This configuration is especially relevant for FC nonlinearities, with minor modifications due to the possible asymmetry of *m*(*t*)^7^,^19^.

In order to complete the analysis, we now assume that the pump pulse has a symmetric Gaussian shape in time, namely, 
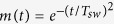
 and that *κ* is purely real as for, e.g., Kerr media; it is also a decent approximation for a free-carrier material due to the quick diffusive self-erasure^7^,^19^. We also assume, for simplicity, 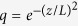. In this case, neglecting third (distortion) term on the LHS of Eqs (2 and 3) (justified by the numerical results, Fig. 3), it can be shown that the backward wave is given by


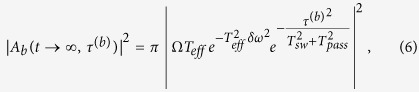


where 
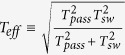
. Thus, the amplitude of the backward wave depends on *T*_*sw*_, *T*_*pass*_ and the relative magnitude of the detuning *δω* with respect to *T*_*eff*_, which is experimentally easy to minimize.

Figure 3 shows that the analytical result (6) is in very good agreement with the numerical results across a wide range of parameters. The analytical result also reconfirms that the temporal duration of the backward wave is indeed substantially shorter than the duration of the input signal, thus, giving a closed-form formula to the amount of *spectral inheritance*. In particular, the duration of the backward pulse is seen to be limited to the longer time-scale in the system, be it either the effective switching time *T*_*sw*_ or the pass time *T*_*pass*_, as expected; Indeed, otherwise it is impossible to explain the appearance of the additional frequency components which do not appear in the forward signal wave. The temporal duration of the backward wave becomes longer than that longest time-scale once the two time-scales (*T*_*sw*_ & *T*_*pass*_) become comparable.

In order to demonstrate the wide range of possible applications of spectral inheritance, we now provide two additional examples. Figure 4 demonstrated spectral inheritance for a much longer interaction, obtained from solving Eqs (2 and 3) numerically. Here, we assumed that a semiconductor (specifically, amorphous silicon waveguide with embedded metal nanoparticles replaces the silica waveguide such that the pump pulses generate a periodic temperature pattern, which, in turn, modifies the semiconductor refractive index^20^. Accordingly, higher efficiencies, up to several tens of percent, are achievable with weaker pump powers; these efficiencies are comparable to those achievable with an out-of-plane configuration of TBGs^3^. Moreover, it is seen that for these conditions, FC absorption hardly affects the efficiency of the scheme. This demonstrates the generation of pulses at so far difficult to access durations of tens to hundreds of picoseconds. As before, the analytical result (6) gives a good prediction of the numerical results, in fact, even beyond its formal limit of validity. Naturally, the theory (6) overestimates the efficiency as the efficiency gets close to 100%.

As a final example, we demonstrate spectral inheritance from a *short* pulse of few picoseconds duration via FC-based diffusive switching in a semiconductor waveguide^7^,^19^. We now assumed a resonantly pumped uniform silicon waveguide relevant parameters are taken from the literature^21^,^22^ such that the pump pulses generate a high density plasma of electron-hole pairs, which, in turn, modifies the semiconductor refractive index^7^,^21^,^22^. Here, the subpicosecond pump and diffusion time give rise to an effective picosecond switching time *despite* the overall nanosecond scale FC recombination time. The CMT solution (Fig. 5) shows that the short TBG can make the generated backward pulse several times shorter than the incoming forward pulse (up to 4-fold shorter in the examples shown; more substantial shortening is also possible at the cost of lower total coupling efficiency); the opposite is also possible (not shown). Moreover, the backward pulse *total* relative power can reach substantial values (>10%) due to the strength of the FC nonlinearity, again, comparable to efficiencies achievable in a similar setup^3^. At the same time, the forward pulse becomes slightly temporally broader. For even stronger perturbations, we observe the formation of a more complicated backward pulse, with increasing number of side lobes in its trailing edge, while the forward wave develops a deep minimum where the backward pulse was extracted; under this condition, FC absorption is not negligible anymore. This example substantially extends the scope of the concept of spectral inheritance by demonstrating short pulse shaping capabilities at high efficiency. Further investigation of this complex dynamics is deferred to a future study.

In summary, we have studied in detail various examples of the complex wave mixing process ensuing from the interaction of pulses of different spatio-temporal extents, and provided a systematic description and interpretation of the otherwise difficult to predict outcoming pulse characteristics in terms of Transient Bragg gratings (TBGs) and *spectral inheritance*. In comparison to the many previous studies of TBGs, which implemented TBGs in bulk and focussed on using them for the measurement of material properties such as diffusion and recombination times, we focus here on the wave physics aspects, specifically, on the ability to generate short backward pulses and the ability to tailor their shape, and on a somewhat more application-appealing configuration of a waveguide. Our approach may open the way to new control levels on the spatio-temporal profiles of short pulses, including to negative frequencies^23^ and very different frequencies^2^, or even to the realization of intricate Fano resonances. On the applicative perspective, our approach can be implemented in a variety of materials, including dielectrics, semiconductors, or even metals and graphene waveguides^24^ with nanosecond sources which are available at a wide range of wavelengths. The efficiencies offered by our approach are not much lower in comparison to those in fiber oscillators, and the simplicity and flexibility in terms of duration and wavelength may open up new opportunities. Our approach can also be used as a seed for later amplification stages towards high power laser applications.

## Additional Information

**How to cite this article**: Sivan, Y. *et al.* Nonlinear wave interactions between short pulses of different spatio-temporal extents. *Sci. Rep.* **6**, 29010; doi: 10.1038/srep29010 (2016).

## Figures and Tables

**Figure 1:**
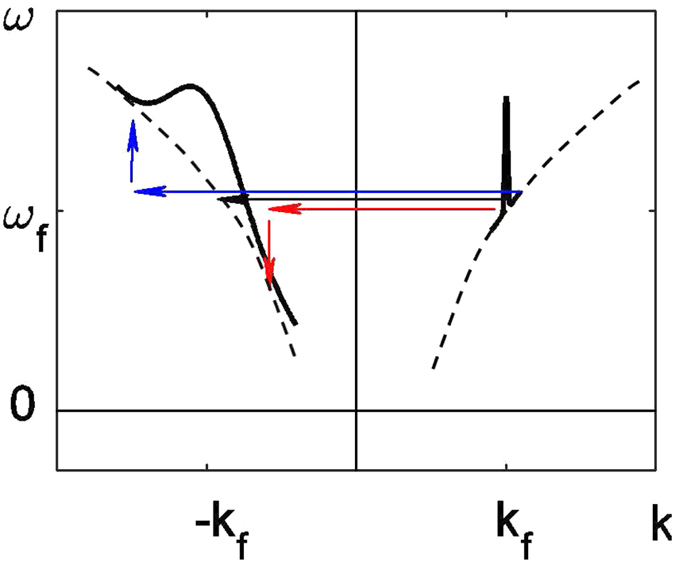
Schematic illustration of *spectral inheritance* - the extraction of a spectrally-wide backward propagating pulse from an initially forward propagating spectrally-narrow pulse - based on transitions between dispersion curves. The horizontal arrows represent momentum that is transferred to the incoming photon from the pump photons, while the vertical arrows represent energy transferred to it. The combination gives rise to a continuum of oblique transitions between the two dispersion branches that in turn result in a spectrally-broad backward pulse.

**Figure 2:**
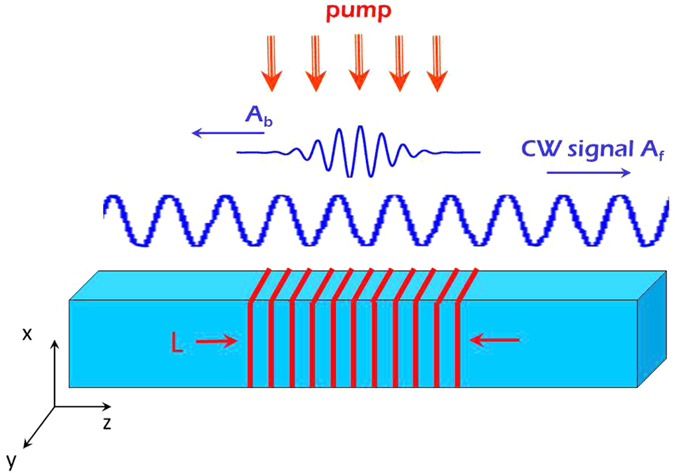
An implementation of the scheme described in Fig. 1. An incoming signal *A*_*f*_ propagates in a uniform waveguide (blue). The periodically-patterned pump pulse, generated by interference of two obliquely incident pulsed beams, traverses the waveguide perpendicularly and generates an *L*-long, *periodic* perturbation of the optical properties (red), chosen such that the induced stop-gap captures the incoming signal pulse and causes the generation of a short backward pulse *A*_*b*_.

**Figure 3:**
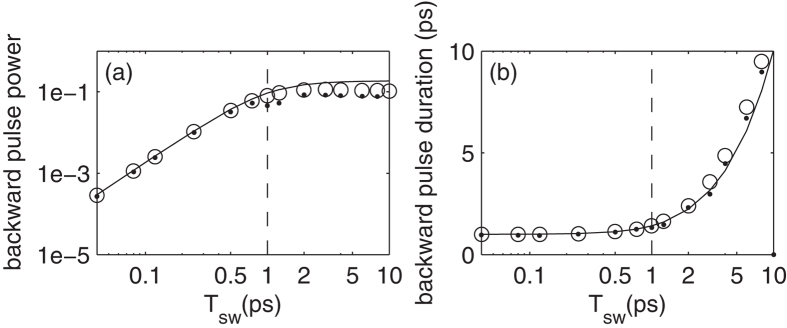
(**a**) Backward pulse (normalized) peak power and (**b**) duration for *T*_*pass*_ = 1 ps (vertical dashed line) as a function of the switching time *T*_*sw*_ for a Gaussian-shaped pump pulse in time and space with *n*_*wg*_ = 1.503, a 2 *μ*m wide in a single mode silica waveguide with *n*_*eff*_ = 1.481 at *λ*_*f*_ = 2 *μ*m, a pump wavelength of 1.5 *μ*m and intensity corresponding to Δ*n*_*wg*,*m*_ ≡ *n*_2_*I* = 2 · 10^−3^. Good agreement is found between the FDTD numerical simulations (dots), CMT simulations (2)-(3) (circles) and the analytic solution (6) (solid line).

**Figure 4:**
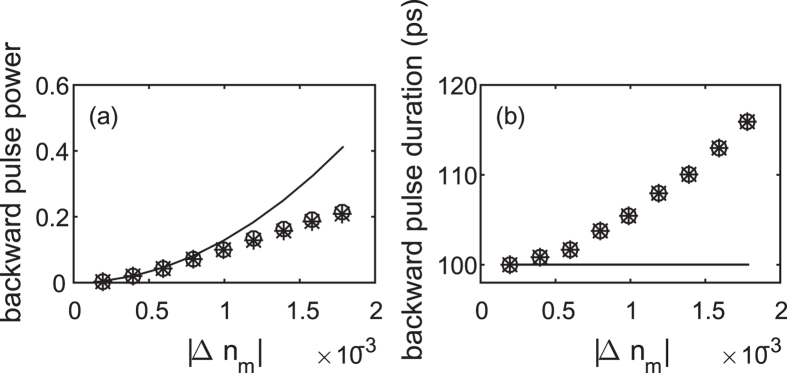
Same as Fig. 3 for *T*_*sw*_ = 100 ps and *T*_*pass*_ = 1.67 ps, *λ*_*pump*_ = 400 nm, *λ*_*f*_ = 1.55 *μ*m. CMT results shown as a function of the refractive index change Δ*n*_*m*_, and compare the lossy case (FC nonlinearity in a silicon waveguide^7^; stars) with the loss-free case (silica waveguide; circles).

**Figure 5:**
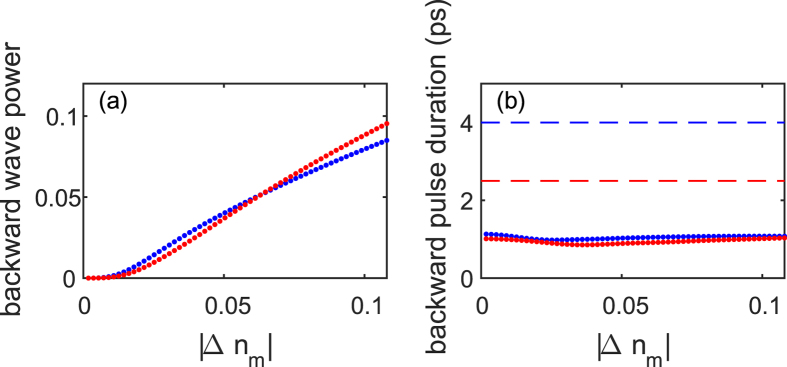
Same as Fig. 4 for the diffusive switching scheme^7^. (**a**) Backward pulse (normalized) *total* power and (**b**) duration for *n*_*wg*_ = 2.5, *T*_*sw*_ ≈ 750 fs, *T*_*pass*_ = 150 fs; data shown for *T*_*f*_ = 4 ps (blue) and *T*_*f*_ = 2.5 ps (red).
